# Clinical Presentation, Diagnostic Delays, and Treatment Outcomes in Postural Orthostatic Tachycardia Syndrome (POTS): An Observational Case Series Study in a Single-Centre District General Hospital

**DOI:** 10.7759/cureus.97581

**Published:** 2025-11-23

**Authors:** Deeya Chaudhury, Naufel Atia, Nonyelum Obiechina, Aftab Gill, Atef Michael, Kavya Sampathy

**Affiliations:** 1 Medicine, University Hospitals of Derby and Burton NHS Trust, Burton, GBR; 2 General Medicine, Queen's Hospital Burton, Burton, GBR; 3 Geriatrics, University Hospitals of Derby and Burton NHS Trust, Derby, GBR; 4 Cardiology, Queen's Hospital Burton, Burton, GBR; 5 Geriatrics, Russells Hall Hospital, Dudley, GBR; 6 Internal Medicine, University Hospitals of Derby and Burton NHS Trust, Burton, GBR

**Keywords:** autonomic nervous system dysfunction, dysautonomia, head up tilt table test, orthostatic intolerance, postural orthostatic tachycardia syndrome (pots)

## Abstract

Background:  Postural orthostatic tachycardia syndrome (POTS) is a heterogeneous disorder of autonomic regulation characterised by unexplained orthostatic tachycardia in the absence of postural hypotension. POTS is a complex and challenging diagnosis owing to the non-specific nature of the presentations, which frequently overlap with other medical conditions. There is limited availability of data and research describing the spectrum of clinical presentations, diagnostic pathways, comorbidities, and management outcomes. This study aims to describe the above in a single-centre district general hospital setting.

Methods:  We conducted a retrospective case series study of 37 patients diagnosed with POTS at Queen’s Hospital Burton (QHB) between August 2023 and August 2024. We used electronic health records to acquire relevant data. This included demographics, presenting complaints, associated conditions, time to diagnosis, specialist involvement, management strategies, and treatment outcomes. 'Time to diagnosis' was defined as the period between the first symptom onset and obtaining a confirmed diagnosis with a positive tilt-table test. Microsoft Excel (Redmond, USA) was utilised for descriptive statistical analysis.

Results and conclusion:  The cohort was predominantly female (n = 36, 97%) with a mean age of 28.2 years (SD, 8.3; range, 18-48). The most common presenting complaints were presyncope (49%) and presyncope with syncope (41%). These were often associated with palpitations and chest discomfort. Systemic conditions coexisting with POTS included anxiety/depression, hypermobility spectrum disorders (notably Ehlers-Danlos Syndrome), fibromyalgia, autoimmune diseases, and migraine. The median time to diagnosis was one year (IQR 1-4). However, delays of up to 20 years were observed. Speciality referrals involved cardiology (65%), neurology (13%), and internal medicine (10%). Management strategies included non-pharmacological therapy alone (19%), additionally pharmacological monotherapy (62%), and combination therapy with multiple drugs (19%). Symptomatic improvement was reported in 65% overall, with the highest rates observed in the multi-drug therapy group.

This study highlights the demographic profile, burden of comorbidities, and diagnostic challenges in patients with POTS. Our single-centre study has demonstrated meaningful progress towards reducing the average time to diagnose POTS with varying treatment outcomes across therapeutic strategies. Multi-drug therapy in conjunction with non-pharmacological therapy proved to be the most efficacious in this cohort. These findings emphasise the importance of early recognition, streamlined referral pathways, and the need for further large-scale multi-centre research into patient-tailored, evidence-based management of POTS.

## Introduction

Postural orthostatic tachycardia syndrome (POTS) is a spectrum of conditions affecting the autonomic regulation of the body, resulting in orthostatic intolerance, leading to a significant increase in the heart rate (>=30 bpm or HR >=120 beats/min) within 10 minutes of standing up from a recumbent position, notably in the absence of any orthostatic hypotension (>20 mmHg drop in SBP) [1,2].

The diagnosis of POTS is often challenging due to the diverse nature of presentation with a high variability and considerable overlap with other cardiovascular, neurological and systemic conditions [3]. Patients present with a constellation of symptoms like palpitations, light-headedness, dizziness, pre-syncope and syncope, leading to a significant impairment in the quality of life [3]. It often leads to a prolonged time to diagnosis involving multiple speciality referrals and delay in commencing appropriate management  [3-5].

The terminology of postural orthostatic tachycardia (POTS) was first mentioned in medical literature in an article published in 1993 by Low et al. [6] and classed as a disorder of the autonomic system. Our comprehension of the underlying pathophysiology of this multifactorial disease has been quite limited and poorly understood so far. The consensus definition of POTS was first established in 2011 by the American Autonomic Society, the European Federation of Autonomic Societies, the Autonomic Research Group of the World Federation of Neurology and the Autonomic Disorders section of the American Academy of Neurology and was published by Freeman et al. [7] and subsequently in 2015 POTS was again defined by the Heart Rhythm Society, in collaboration with representatives from the American Autonomic Society (AAS), the American College of Cardiology (ACC), the American Heart Association (AHA), the Asia Pacific Heart Rhythm Society (APHRS), the European Heart Rhythm Association (EHRA), the Paediatric and Congenital Electrophysiology Society (PACES), and the Latin American Society of Cardiac Pacing and Electrophysiology (SOLAECE) and this work was published by Sheldon et al. [1].

On adopting an upright position, the human body shifts approximately 500 ml of blood from the thorax to the lower abdominal, pelvic and lower limb veins, or the capacitance vessels [8]. This venous pooling may lead to a decreased venous return to the heart and cause a drop in cardiac output [2]. The transient lowering of blood pressure during upright posture in healthy individuals causes a reflex response of tachycardia, which is usually just over 100 beats/minute [9]. In POTS, due to the various mechanisms, this physiological response is exaggerated, and the tachycardia is extreme due to the inadequate peripheral and splanchnic vasoconstriction. 

Researchers have proposed various classifications for POTS. According to Grubb (2008), the most clinically significant division is into primary and secondary POTS [10]. Primary POTS occur independently, without association with other diseases, while secondary POTS is linked to underlying conditions such as diabetes mellitus, amyloidosis, sarcoidosis, autoimmune disorders like lupus or Sjogren’s syndrome, chemotherapy, or post-viral illnesses including COVID-19 [10].

Various mechanisms have been found to underlie POTS; a considerable overlap has been observed amongst the aetiologies. The aetiology of POTS can be classified as follows [3]: 

Hypovolemic type POTS

Many people with POTS have low blood volumes. Studies have demonstrated a plasma volume deficit of almost 13% in patients with POTS [2]. These patients have comparatively reduced levels of standing plasma renin activity and aldosterone, which can contribute to systemic volume dysregulation [5]. Relative hypovolemia in this case or following either poor fluid intake or loss of fluids due to gastrointestinal disorders can lead to the development of secondary orthostatic intolerance and eventually POTS [5]. 

Hyper-adrenergic type POTS

It is characterised by a high level of circulating norepinephrine during orthostasis; patients demonstrate a tendency to orthostatic hypertension and often complain of extremity tremors, anxiety, migraine and angina-like chest pain [11]. The level of circulating norepinephrine has been demonstrated to be equal to or above 600 pg/mL [5]. Mast cell activation syndrome (MCA) is a clinical entity which is linked to POTS and is considered a secondary hyperadrenergic subtype of POTS. It is characterised by flushing, headache, dyspnoea, and gastrointestinal symptoms like nausea, vomiting, and diarrhoea [11]. Studies on mast cell activation syndrome utilise the measurement of urinary methylhistamine to diagnose episodes of mast cell activation [12]. Research has shown that the underlying mechanism is the hyperadrenergic form of POTS due to the exaggerated sympathetic pressor response during the Valsalva manoeuvre and an increase in blood pressure on orthostasis in these patients [12].

Neuropathic type POTS

This subtype of POTS is presumed to occur due to the impaired sympathetically mediated peripheral vasoconstriction, leading to venous pooling in the lower limbs. This sympathetic denervation can cause an impairment of sweating in the lower extremities and reduced release of norepinephrine [5]. This type is neuropathic in origin, as here venous pooling occurs in spite of no defect in the venous capacitance vessels. It can be confirmed by the measurement of blood pressure profile during the head-up tilt table test [5]. Small fibre neuropathy in the lower limbs is the primary reason for decreased release of norepinephrine, especially in the lower limbs [13]. Clinically, this can manifest as acrocyanosis due to the peripheral pooling of blood [13].

Deconditioning

Studies have shown that bed rest or deconditioning can contribute independently to the development of POTS, regardless of the underlying mechanism [5]. Longer periods of physical inactivity can lead to cardiac muscular atrophy, reduction in stroke volume and overall hypovolemia [14]. Impairment of reflexes like the vasoconstrictor baroreceptor reflex and the vestibulo-sympathetic reflex is also a contributing mechanism [5]. 

Current literature indicates considerable overlap among the underlying mechanisms of POTS, prompting a shift in our understanding of the syndrome. This may account for the observed efficacy of combination therapy, employing both non-pharmacological interventions and a combination of pharmacologic agents targeting different pathways, over monotherapy in certain patient populations. 

The existing research on POTS comprises data collected and reported from tertiary centres or national surveys conducted by specialised POTS centres. This study was undertaken in a single-centre district general hospital to assess the clinician's understanding of POTS, evaluate the time to diagnosis, and examine management strategies in a resource-limited healthcare setting.

Aims and objectives

The objectives of this single-centre retrospective case series were: A) to identify time to diagnosis and co-morbid conditions in a cohort of POTS patients. B) To assess treatment modalities and outcomes of these patients.

## Materials and methods

Study design and setting  

We conducted a retrospective case series at Queen’s Hospital Burton, a district general hospital in the United Kingdom, reviewing patients diagnosed with Postural Orthostatic Tachycardia Syndrome (POTS). 

*Inclusion Criteria * 

All patients meeting the standardised definition of POTS, whose diagnosis was confirmed with positive tilt table testing between August 2023 and August 2024. A tilt table test was considered positive if the heart rate increased by >=30 bpm or the heart rate was >= 120 bpm within 10 minutes of standing up from a recumbent position, notably in the absence of any orthostatic hypotension (>20 mmHg drop in SBP). The FINAPRES NOVA Plus Syncope System was used for tilt table testing.  

Exclusion Criteria 

Patients with insufficient data regarding presentation, management, past medical history, and treatment outcomes as per electronic health records. Three patients were excluded from data analysis based on these exclusion criteria.  

*Statistical Analysis* 

Microsoft Excel was utilised in data analysis. Descriptive statistics were used to analyse demographic, clinical, and outcome data. Continuous variables are presented as mean ± standard deviation (SD) or median with interquartile range (IQR), as appropriate. Categorical variables are reported as absolute counts and percentages.  

*Ethical Considerations* 

This study was conducted as a retrospective service evaluation and registered with the clinical governance department at Queen’s Hospital Burton. The results in this publication originated from data obtained during an audit process. No intervention was performed during the audit. Patient data was anonymised, and formal ethical approval was not required. 

*Data Collection*  

Anonymised data were acquired from electronic health records using a standardised proforma. Variables included: demographics: age, sex, clinical features: presenting complaints and associated symptoms, past medical history: co-morbidities classified as psychiatric, connective tissue, autoimmune, neurological, chronic pain, or unrelated conditions, diagnostic pathway: time from symptom onset to diagnosis, specialists involved prior to confirmation of diagnosis, management: non-pharmacological measures (e.g., increased salt/fluid intake, compression stockings, exercises), pharmacological monotherapy, and combination pharmacotherapy, treatment outcomes on follow-up: measured using subjective description by the participants with documented symptomatic improvement on follow-up, no change, or worsening symptoms following management.  

*Diagnostic Modality*  

Head-up tilt table testing (with passive standing) was utilised as the diagnostic method. The standing angle utilised was 70 degrees from horizontal. The FINAPRES Nova (Enschede, the Netherlands) machine with non-invasive beat-to-beat monitoring of blood pressure was used. A backup blood-pressure cuff machine was also used for blood pressure monitoring as a part of quality control. 

## Results

Demographics   

A total of 37 patients with a confirmed diagnosis of postural orthostatic tachycardia syndrome (POTS) were identified at Queen’s Hospital Burton, between August 2023 and August 2024. The cohort was predominantly female (n = 36, 97%) with a single male patient (3%). The mean age at diagnosis was 28.2 years (SD 8.3), with a median age of 27 years (range 18-48).

Presenting symptoms

The cohort studied consisted of 37 patients, with overlapping symptoms among many of them. The most frequent presenting symptom was pre-syncope, occurring in 33 patients, either alone or alongside other symptoms such as palpitations, syncope, chest pain, and shortness of breath. Palpitations were the second most common symptom, reported by 17 patients. Among those, two were pregnant patients who experienced palpitations alone. Syncope was observed in a similar number of patients (n=17), often in conjunction with other issues. Notably, 15 patients experienced both pre-syncope and syncope. Other symptoms included isolated syncope (n=2), chest pain (n=5), shortness of breath (n=2), visual disturbances (n=2), tinnitus (n=1), and nausea (n=1). 

Associated comorbidities   

Comorbidities were commonly present within the cohort, with only 11 patients (30%) having no past medical history. Sixteen patients (43%) had conditions deemed non-associated with POTS. Psychiatric, connective tissue, autoimmune, neurological, and chronic pain disorders were frequently observed.  

Psychiatric and Functional Disorders

Anxiety and/or depression were present in seven patients, functional neurological disorder in three, and non-epileptic seizures in one.  

Connective Tissue and Pain Syndromes

Hypermobility spectrum disorder was present in seven patients, including two patients who had Ehlers-Danlos syndrome type 3, fibromyalgia in five, and chronic pain syndrome in two.  

Neurological Conditions

Migraine was reported in three patients, chronic fatigue syndrome in one, and multiple sclerosis in one.  

Autoimmune Disease

Documented in five patients, comprising Crohn’s disease (n = 2), Sjogren’s syndrome (n = 1), psoriatic arthritis (n = 1), and multiple sclerosis (n = 1).  

Other Conditions

Right bundle branch block (n = 1) and a small patent foramen ovale (n = 1).  

Interestingly, two patients reported symptoms following COVID-19/viral Illness. 

Overall, anxiety/depression, hypermobility spectrum disorders, fibromyalgia, and autoimmune diseases were the most prevalent associated conditions in accordance with the broader existing literature related to POTS.

Time to diagnosis   

**Figure 1:**
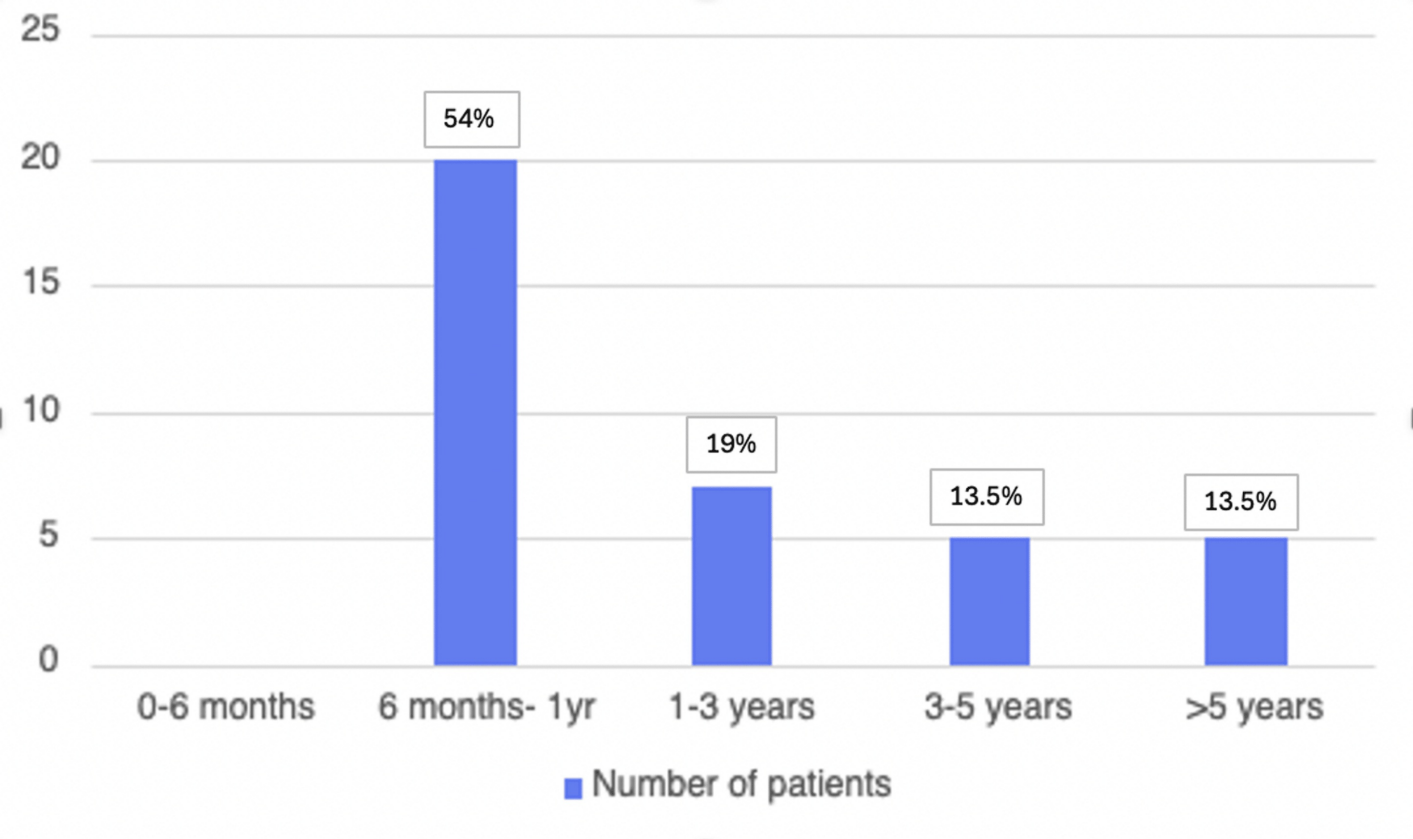
Time taken to diagnose the patients with POTS

The period from initial symptom onset to formal POTS diagnosis varied significantly among the patient cohort: The median time from symptom onset to confirmed diagnosis was one year (interquartile range [IQR] 1-4 years). The mean time to diagnosis was 2.9 years from the time of symptom onset (SD 3.5), with a range of 1-20 years. Twenty patients (54%) were diagnosed with POTS within 6 months to 1 year of symptom onset, seven patients were diagnosed within 1-3 years (18.9%), five patients (13.5%) within 3-5 years, and five patients (13.5%) experienced delays of five years or more; only one of these five patients was an outlier who experienced a diagnostic delay of 20 years from the onset of her symptoms (Figure 1).  The data regarding 'time to diagnosis' has been demonstrated in Figure 1.

Clinical teams involved in diagnosis

Cardiologists were the most frequently involved specialists in diagnosing and managing POTS (65%) compared to 13% by neurologists and 10% by the acute medical team. 

Diagnostic modality

**Figure 2:**
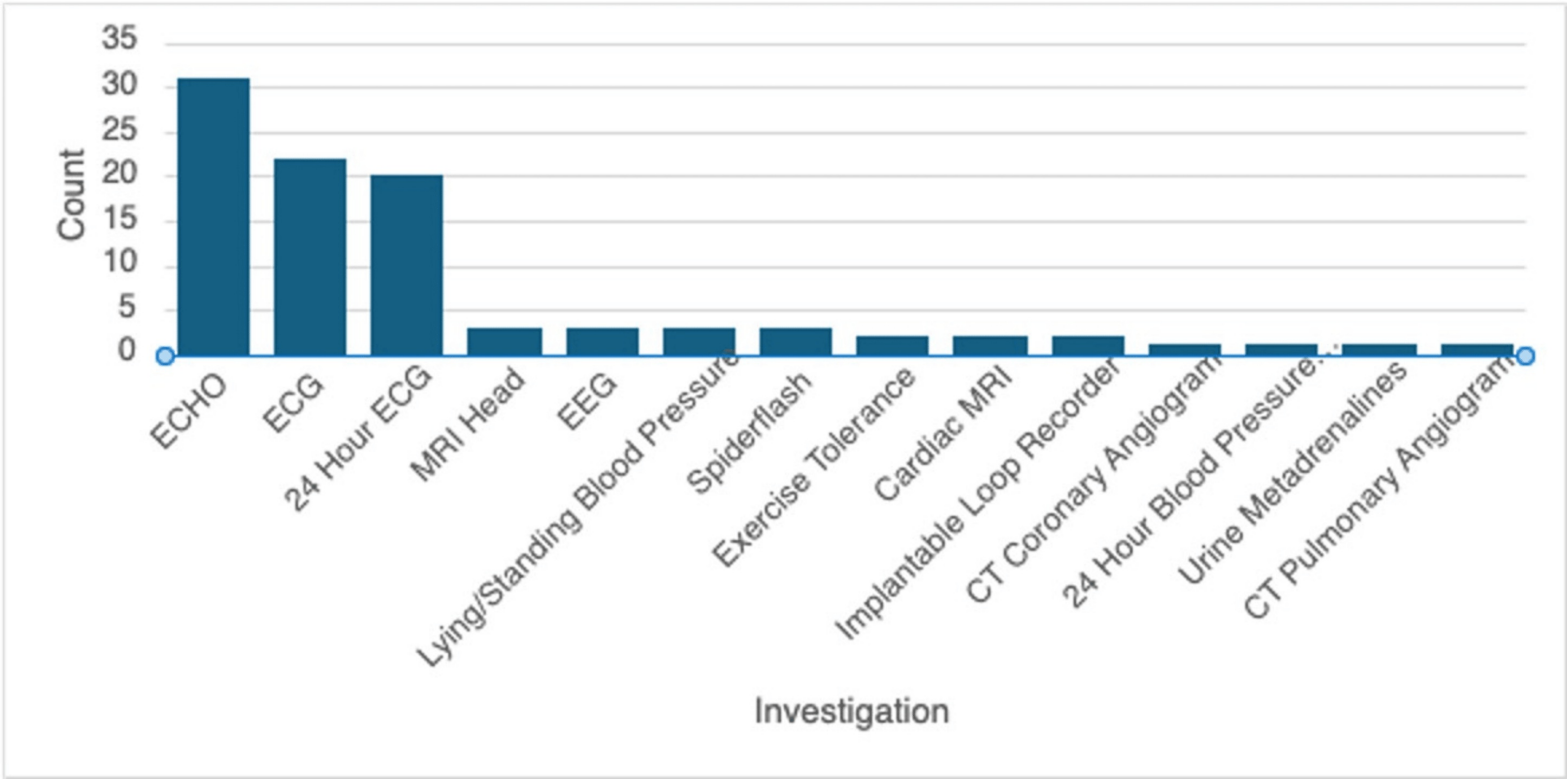
Diagnostic modalities used prior to tilt-table testing

The different investigations performed prior to reaching the diagnosis with the tilt-table test in our setting are demonstrated in Figure 2. Amongst the pre-tilt table investigations, echocardiogram (ECHO), electrocardiogram (ECG), and ambulatory ECG were the most common diagnostic tools in the workup to diagnose POTS. ECHO was the most commonly used diagnostic modality (n=31, 84%), with ECG being the second most common (n=22, 59%). The third most common investigation was 24-hour ECG monitoring (n=18, 49%). Generally, all three investigations were performed together to arrive at a diagnosis. Most of the investigations performed prior to tilt table testing did not show any abnormalities (n=87, 92%), with a minority of the tests coming back with abnormalities (n=8, 8%). Shortened PR interval (n=4, 50%) accounted for most of the abnormalities detected, with impaired left ventricular function (n=2, 25%) present in two patients.

Alternative diagnosis given

In 30 patients (81%), no alternative diagnoses were considered prior to the diagnosis of POTS, whereas seven people (19%) were mislabelled as having vasovagal syncope, epilepsy, anxiety, and other conditions. 

Treatment options   

Treatment options in our study center included non-pharmacological measures alone as the first line of management for all patients diagnosed with POTS, followed by the addition of pharmacological monotherapy as the second line and the addition of further drugs as a part of combination therapy in case of severe, persistent symptoms or inadequate clinical response.

Amongst the 37 patients studied, seven patients (19%) were managed conservatively with lifestyle modifications like increased fluid and salt intake, reduction of alcohol and caffeine intake, eating smaller meals, and using compression garments. Pharmacological monotherapy was initiated in 23 patients (62%), and seven patients (19%) underwent multi-drug therapy. The most common agents used in monotherapy were Ivabradine (n=12, 52%) and Bisoprolol (n=8, 35%). Other drugs used included fludrocortisone, midodrine, and propranolol.

Amongst the combination drug regimens, Ivabradine with Midodrine was the most common option (n=3, 43%), followed by other combinations such as Bisoprolol, Ivabradine, and Midodrine (n=2, 29%); Fludrocortisone and Ivabradine (n=1, 14%); and Fludrocortisone and Midodrine (n=1, 14%).

Treatment outcomes   

**Figure 3:**
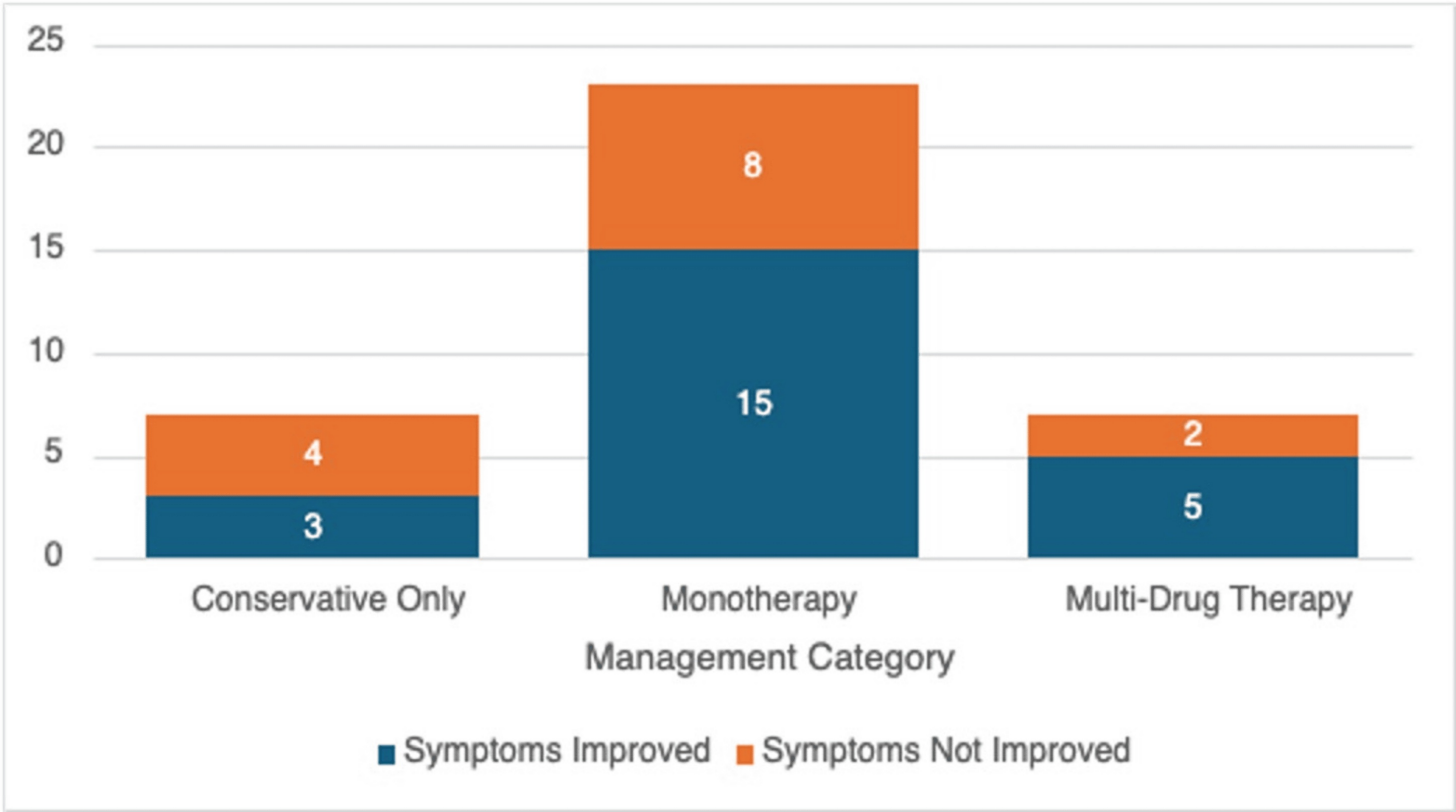
Treatment outcomes for management of POTS patients

A comparison of symptom resolution as per patient-reported outcomes at a six-month follow-up post-initiation of therapy has been outlined in Figure 3. This comparison demonstrated that, along with non-pharmacological therapy, a multi-drug regimen resulted in a symptomatic improvement in five out of seven patients (71%), followed by monotherapy, which was effective in 15 out of 23 patients (65%), while non-pharmacological conservative measures alone benefited three out of seven patients (42%).

**Figure 4:**
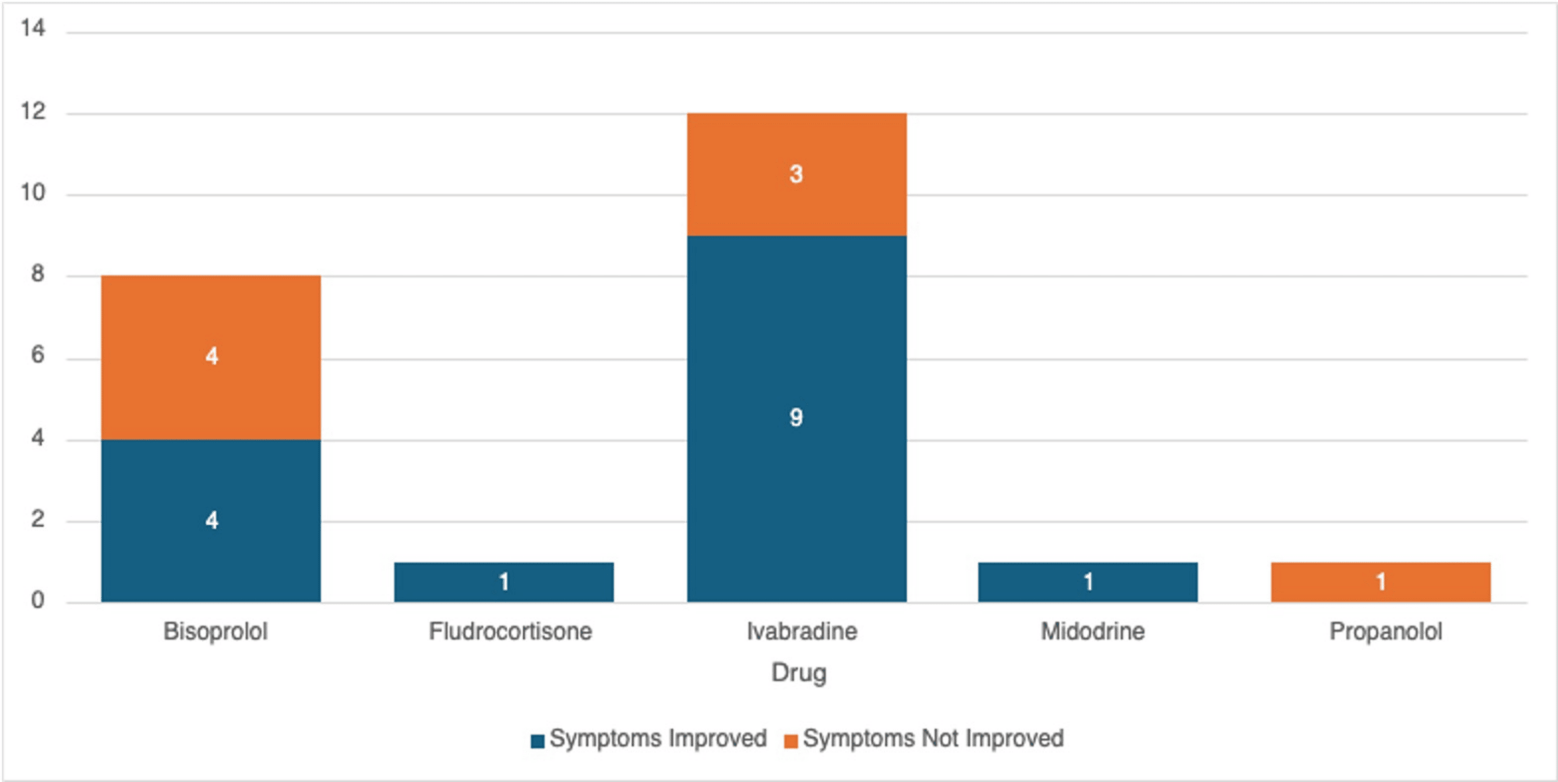
Treatment outcomes for drugs used in monotherapy

The effect of monotherapy post-treatment with each drug individually has been showcased in Figure 4. A total of 23 patients underwent monotherapy. This included the drugs bisoprolol, fludrocortisone, ivabradine, midodrine, and propranolol. Both fludrocortisone and midodrine were used as monotherapy in one patient each and resulted in symptomatic improvement in both these patients; however, the interpretability of these findings is limited by the small sample size (n=1 for each treatment). Ivabradine showed symptom improvement in nine patients out of 12 patients, followed by bisoprolol, which showed an improvement in four out of eight patients. Propranolol had no reported improvement on symptoms; however, this was used only in a single patient. 

**Figure 5:**
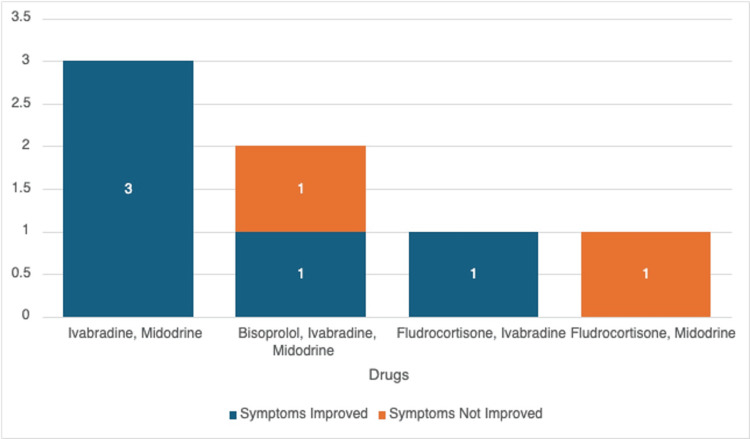
Multi-drug therapy outcomes

Figure 5 illustrates the different multi-drug therapeutic combinations utilized in our hospital setting for the treatment of POTS and the improvement of symptoms in response to each of these therapeutic interventions. For patients receiving multi-drug therapy, ivabradine and midodrine yielded a 100% symptom improvement rate (3/3). Similarly, the combination of fludrocortisone and ivabradine also demonstrated complete symptom resolution; this finding should be interpreted with caution, given that this finding is based on a single patient (n=1). Triple therapy comprising bisoprolol, ivabradine, and midodrine resulted in symptom improvement in 50% of cases (1/2). The combination of fludrocortisone and midodrine did not lead to symptom improvement, though this observation is limited by the small sample size (n=1). 

## Discussion

In this observational case series, we have tried to provide a comprehensive description of the clinical characteristics, diagnostic trajectory, comorbidities, management strategies, and treatment outcomes in a cohort of patients with postural orthostatic tachycardia syndrome (POTS) diagnosed at a single district general hospital in the UK. 

Several key findings of this case series consisted of demonstrating the prevalence of POTS among younger females and a high incidence of associated systemic conditions, including hypermobility and autoimmune disease. It also showed an improved, yet still substantial, delay to diagnosis in a significant proportion of patients compared to some of the previous studies [4]. There were variable treatment outcomes with evidence of greater benefit from concomitant multi-drug therapy with conservative measures as compared to conservative measures alone or with single-agent use.  It is worth noting that these are not head-to-head trials, and therefore, the findings need to be interpreted with caution. 

Strengths of the study

This study, despite a relatively small sample size, aligns with emerging patterns in wider POTS literature, such as female predominance, a multitude of symptoms and presentations, therapeutic interventions, and treatment outcomes. In this study, we note outcomes that are consistent with the trends reported by established studies [4], and in our study setting, we have shown a reduction in time taken to diagnosis and less mislabelling of POTS as alternative diagnoses.

Limitations of the study

This study has several limitations. Its retrospective design and single-center setting limit generalizability. Data were dependent on the accuracy and completeness of electronic health records. Treatment outcomes were based on patient-reported improvement and clinical documentation rather than standardized quality-of-life measures. Furthermore, the relatively small sample size precludes detailed subgroup analysis of treatment response by comorbidity or demographic variables. Treatment outcomes should be interpreted with caution since these treatments are not head-to-head clinical trials.

Female predominance

It is widely demonstrated that the ratio of affected females to males in POTS is 5:1. A typical POTS patient would be a young female in the age of 20-40 years [9]. This high female preponderance can result in a significant impairment to their quality of life during their reproductive years and can be debilitating for some. Our study participants also included two pregnant women, for whom pregnancy caused the symptoms of POTS.

Co-morbid conditions

POTS is well known to be associated with several conditions, such as chronic fatigue syndrome and joint hypermobility, specifically Ehlers-Danlos syndrome type III, which have been linked to extra-articular manifestations such as dysautonomia and POTS [15]. 

Long COVID

The COVID-19 pandemic in 2019 has presented new challenges to the medical and scientific communities. POTS is a clinical entity with a surge of cases noted in the recovery period of prolonged COVID illness. It is difficult to determine whether new symptoms occurring in this subgroup of patients post-COVID illness are related to a separate entity of POTS or whether they are due to ‘Long COVID.’ Therefore, if patients present with symptoms like brain fog, chronic fatigue syndrome, tachycardia, or clinical POTS in the 12 weeks following a COVID infection, it is classified as ‘Long COVID POTS’ [16]. The evidence for the mechanism to explain this association is scarce, but some studies have suggested involvement of the renin-angiotensin aldosterone system impacting the blood volume due to the dependency of the SARS-COV-2 virus on the receptor ACE-2 and TMPRESS for its entry into the host cell [17]. 

Autoimmune disorders

The term "secondary POTS" is often reserved for the subtype associated with a chronic disease or autoimmune syndrome. About 20% of patients with an autoimmune disease have POTS [18]. Amongst them, chronic diabetes mellitus is the most common disease related to POTS [19]. Frequent associations include Hashimoto’s thyroiditis, Sjogren’s syndrome, antiphospholipid syndrome, systemic lupus erythematosus (lupus), and rheumatoid arthritis [19]. In our study, patients also had autoimmune diseases, which were Sjogren’s syndrome, Crohn’s disease, psoriatic arthritis, and multiple sclerosis. Research is underway to establish a link between certain autoantibodies, such as G-protein-coupled adrenergic receptors and muscarinic cholinergic receptor antibodies found in elevated levels in POTS, which corresponds to the theory that POTS may have an immune-mediated pathogenesis [19].

Psychiatric associations and mental health disorders

A small category of POTS patients with co-existing Ehlers-Danlos syndrome type III (hypermobility type) also present with a spectrum of psychiatric illnesses comprising anxiety disorders, particularly panic disorders and agoraphobia [20]. 

Time to diagnosis

As compared to observational studies and surveys performed in the last decade, there has been a paradigm shift in the understanding of the nature of postural orthostatic tachycardia syndrome. In 2015, a large survey consisting of 779 respondents carried out by members of POTS UK, published in March 2016, established that the mean time to diagnosis was 3.7 years [21]. Now, there is a greater awareness among clinicians in primary care as well as point-of-care physicians regarding the inconspicuous variety of presentations. It is reflected in the increased availability of head-up tilt table testing in multiple centers and improved multi-specialty referral pathways at an early stage. Our study, conducted in a single-center setting, has demonstrated this, with over 50% of our patients being diagnosed within one year of symptom onset. This can set a foundation for further randomized clinical trials to comprehend the full extent of improved diagnoses.

Selecting an appropriate therapeutic agent to treat POTS

The choice of therapeutic agent is variable, generally in people with POTS, and depends on the presenting symptom and the primary suspected underlying pathophysiology of POTS. Not all therapeutic interventions benefit every POTS patient [2].

Central sympatholytic drugs like clonidine and methyldopa have been shown to positively impact the symptoms in the hyperadrenergic variant of POTS but remain of limited use in the neuropathic variant [2].

Beta-adrenergic antagonists like bisoprolol and low-dose propranolol control the tachycardia and often provide symptomatic relief in POTS [22]. Selective sodium channel blockers (funny current), like Ivabradine, which target the cardinal manifestation of tachycardia in POTS, have shown effectiveness in low doses in some trials [23].

In neuropathic POTS, the loss of peripheral vascular resistance due to the sympathetic denervation, especially in the lower limbs, can be addressed with the use of vasoconstrictors like midodrine [13,24]. 

Our study corroborates with the established treatments in POTS with different agents. In our study, we have noted that Ivabradine is superior in terms of symptomatic improvement as both a monotherapy and in combination with other drugs. As monotherapy, it resulted in symptom resolution in 75% of cases, while in combination with midodrine, it was efficacious in all three patients who received this. Comparing this data to established clinical trials showcasing the effects of ivabradine as a single therapy, specifically in hyperadrenergic POTS, as demonstrated by Taub et al. [25] and McDonald et al. [23], this study adds evidence to the fact that ivabradine monotherapy is increasingly being used in POTS, with favorable outcomes of reducing the heart rate during orthostasis while maintaining the same blood pressure. 

Often patients are trialed on a multi-drug approach due to non-response from monotherapy or the need to target different co-existing pathologies. We can use midodrine in addition to ivabradine if there is an overlap of symptoms with neuropathic POTS [24]. Our study shows that a combination of Ivabradine with Bisoprolol and Midodrine or Ivabradine with Fludrocortisone was both effective in symptom resolution. 

Burden of living with POTS 

The chronic nature of POTS can make it a debilitating disease for the individuals affected, as they are confined to wheelchairs or become bedbound due to their inability to tolerate any form of orthostasis. Combined with the non-postural symptoms of gastrointestinal and neurological diseases like brain fog, chronic fatigue, and autoimmune disease burden, it significantly impairs the quality of life for patients. Studies performed by the Mayo Clinic documented symptomatic improvement in 80% of patients on follow-up, with 60% of patients being able to function normally and 90% of patients being able to return to work [26]. In our study, we have observed patient-reported symptomatic improvement, but long-term studies and follow-up studies are needed for further clarification of the findings. 

## Conclusions

This case series highlights the importance of establishing a systematic approach to diagnosing complex conditions like POTS. In a resource-limited district general hospital setting, we have adopted head-up tilt-table testing with noninvasive beat-to-beat monitoring as the diagnostic method and, over a period of two years, have diagnosed 41 patients with postural orthostatic tachycardia syndrome (POTS).

The findings of this case series add strength to the wider existing evidence body regarding demographic preponderance, associated co-morbidities, and treatment options and further reinforce positive outcomes following multi-drug therapeutic options.  First, young women presenting with presyncope, syncope, and unexplained palpitations should prompt consideration of POTS, particularly in the presence of associated conditions such as hypermobility or autoimmune disease. Second, an improvement in time to diagnosis, although in a small cohort, offers a positive direction and emphasizes the need for improved awareness and streamlined referral processes. Finally, while multi-drug regimens may offer superior outcomes for selected patients, this needs further evidence from multi-center randomized controlled trials to establish optimal therapeutic strategies. 

## References

[1] Sheldon RS, Grubb BP 2nd, Olshansky B (2015). 2015 heart rhythm society expert consensus statement on the diagnosis and treatment of postural tachycardia syndrome, inappropriate sinus tachycardia, and vasovagal syncope. Heart Rhythm.

[2] Raj SR (2013). Postural tachycardia syndrome (POTS). Circulation.

[3] Steinberg RS, Dicken W, Cutchins A (2025). US Cardiology Review: Narrative review of postural orthostatic tachycardia syndrome: associated conditions and management strategies. https://www.uscjournal.com/articles/narrative-review-postural-orthostatic-tachycardia-syndrome-associated-conditions-and?language_content_entity=en.

[4] Shaw BH, Stiles LE, Bourne K (2019). The face of postural tachycardia syndrome - insights from a large cross-sectional online community-based survey. J Intern Med.

[5] Benarroch EE (2012). Postural tachycardia syndrome: a heterogeneous and multifactorial disorder. Mayo Clin Proc.

[6] Schondorf R, Low PA (1993). Idiopathic postural orthostatic tachycardia syndrome: an attenuated form of acute pandysautonomia?. Neurology.

[7] Freeman R, Wieling W, Axelrod FB (2011). Consensus statement on the definition of orthostatic hypotension, neurally mediated syncope and the postural tachycardia syndrome. Clin Auton Res.

[8] Goswami N, Blaber AP, Hinghofer-Szalkay H, Convertino VA (2019). Lower body negative pressure: physiological effects, applications, and implementation. Physiol Rev.

[9] Raj SR (2006). The postural tachycardia syndrome (POTS): pathophysiology, diagnosis & management. Indian Pacing Electrophysiol J.

[10] Grubb BP (2008). Postural tachycardia syndrome. Circulation.

[11] Fedorowski A (2019). Postural orthostatic tachycardia syndrome: clinical presentation, aetiology and management. J Intern Med.

[12] Shibao C, Arzubiaga C, Roberts LJ 2nd (2005). Hyperadrenergic postural tachycardia syndrome in mast cell activation disorders. Hypertension.

[13] Mar PL, Raj SR (2020). Postural orthostatic tachycardia syndrome: mechanisms and new therapies. Annu Rev Med.

[14] Levine BD, Zuckerman JH, Pawelczyk JA (1997). Cardiac atrophy after bed-rest deconditioning: a nonneural mechanism for orthostatic intolerance. Circulation.

[15] Rowe PC, Barron DF, Calkins H (1999). Orthostatic intolerance and chronic fatigue syndrome associated with Ehlers-Danlos syndrome. J Pediatr.

[16] Amekran Y, Damoun N, El Hangouche AJ (2022). Postural orthostatic tachycardia syndrome and post-acute COVID-19. Glob Cardiol Sci Pract.

[17] Hoffmann M, Kleine-Weber H, Schroeder S (2020). SARS-CoV-2 cell entry depends on ACE2 and TMPRSS2 and is blocked by a clinically proven protease inhibitor. Cell.

[18] Pena C, Moustafa A, Mohamed AR, Grubb B (2024). Autoimmunity in syndromes of orthostatic intolerance: an updated review. J Pers Med.

[19] Gunning WT 3rd, Kvale H, Kramer PM (2019). Postural orthostatic tachycardia syndrome is associated with elevated G-protein coupled receptor autoantibodies. J Am Heart Assoc.

[20] Yahya AS, Khawaja S (2025). Psychiatrist.com: Psychiatric disorder in postural orthostatic tachycardia syndrome and Ehlers-Danlos syndrome-hypermobility type. Prim Care Companion CNS Disord.

[21] Kavi L, Nuttall M, A Low D (2016). A profile of patients with postural tachycardia syndrome and their experience of healthcare in the UK. Bri Jr Car.

[22] Raj SR, Black BK, Biaggioni I (2009). Propranolol decreases tachycardia and improves symptoms in the postural tachycardia syndrome: less is more. Circulation.

[23] McDonald C, Frith J, Newton JL (2011). Single centre experience of ivabradine in postural orthostatic tachycardia syndrome. Europace.

[24] Miller AJ, Raj SR (2018). Pharmacotherapy for postural tachycardia syndrome. Auton Neurosci.

[25] Taub PR, Zadourian A, Lo HC (2021). Randomized trial of ivabradine in patients with hyperadrenergic postural orthostatic tachycardia syndrome. J Am Coll Cardiol.

[26] Sandroni P, Opfer-Gehrking TL, McPhee BR, Low PA (1999). Postural tachycardia syndrome: clinical features and follow-up study. Mayo Clin Proc.

